# Influenza season 2020–2021 did not begin in Finland despite the looser social restrictions during the second wave of COVID‐19: A nationwide register study

**DOI:** 10.1002/jmv.27048

**Published:** 2021-05-12

**Authors:** Ilari Kuitunen

**Affiliations:** ^1^ School of Medicine University of Eastern Finland Kuopio Finland; ^2^ Department of Pediatrics Mikkeli Central Hospital Mikkeli Finland

**Keywords:** epidemiology, influenza, nonpharmaceutical interventions

## Abstract

The nationwide lockdowns ended influenza seasons rapidly in Northern Hemisphere in Spring 2020. The strategy during the second wave was to minimize the restrictions set for children. Children spread influenza and therefore simultaneous influenza and COVID‐19 surges were feared. The aim of this report is to analyze the epidemiology of influenza season 2020–2021 in Finland. Data for this retrospective register‐based study were gathered from the National Infectious Disease Register, all laboratory‐confirmed influenza cases from August 2017 to March 2021 were included. The positive influenza findings were stratified by age, and incidences per 100 000 persons were calculated. Only 41 influenza A and B cases have been reported in this season from August 2020 to March 2021, which adds up to an incidence of 0.9 per 100 000 person‐years. In the three preceding years, the numbers and corresponding incidences from August to March were 12 461 (282 per 100 000 person‐years) in 2019–2020, 15 276 (346 per 100 000 person‐years) in 2018–2019, and 33 659 (761 per 100 000 person‐years) in 2017–2018. Nonpharmaceutical interventions combined with the lockdown measures interrupted the influenza season in Finland in March 2020. Despite looser restrictions, alongside traveling restrictions and facial masks, failing to prevent the spread of the severe acute respiratory syndrome coronavirus 2 virus, these restrictions have proved to be effective against seasonal influenza.

## 1 BACKGROUND

It has now been a year since the coronavirus disease (COVID‐19) caused by severe acute respiratory syndrome coronavirus 2 (SARS‐CoV‐2) was declared a pandemic on March 11, 2020 by the World Health Organization. Most countries implemented lockdown measures in the spring of 2020. The lockdown strategy was effective for preventing the transmission of the SARS‐CoV‐2 virus, as well as other respiratory pathogens, especially influenza.^1^, ^2^, ^3^ In the summer of 2020, the overall incidence of SARS‐CoV‐2 infection was low in Europe, and many of the lockdown restrictions were lifted. During the second COVID‐19 wave, regional stepwise restrictions were implemented across Finland. Furthermore, wearing facial masks was promoted, but was not made mandatory, and traveling was to be avoided but was not prohibited. Elementary schools and day‐care centers have remained open since May 2020.

Before this COVID‐19 pandemic, the evidence on the impact of nonpharmaceutical interventions on common respiratory diseases was vague. According to a Cochrane review, the effects of wearing facial masks were controversial.^4^ Children play a key role in spreading influenza, but the role of children in the spread of COVID‐19 is still a matter of debate.^5^, ^6^, ^7^ A major worry in Finland before winter 2020–2021 was the possibility of simultaneous surges in influenza epidemics and the COVID‐19 pandemic. Previous reports have described the simultaneous COVID‐19 and influenza infections to be severe.^8^, ^9^ Furthermore, influenza epidemic outbreaks were reported as the restrictions were eased.^10^ In Australia, there was a significant increase in cases of respiratory syncytial viruses as the restrictions were lifted.^11^ The goal in Finland was to keep schools open and restrict the spread of COVID‐19 by implementing the *test*, *track*, and *quarantine* strategy. However, it was possible that influenza could still spread among children. The Finnish Institute of Health and Welfare actively promoted influenza vaccinations, and the vaccinations were all used already in November 2020.

This report describes the epidemiology of influenza A and B infections in Finland during the 2020–2021 winter season.

## 2 MATERIALS

Data for this retrospective cross‐sectional register‐based study were gathered from the National Infectious Disease Register, maintained by the Finnish Institute of Health and Welfare. The register is a daily updated open‐access surveillance system to which all laboratories are mandated by the law on contagious diseases to immediately report all findings on notifiable diseases. The reporting delay is minimal, and the register provides current information. The complete list of notifiable diseases can be found in the register description (https://thl.fi/en/web/infectious-diseases-and-vaccinations/surveillance-and-registers/finnish-national-infectious-diseases-register).

For this study, all laboratory‐confirmed influenza cases from August 2017 to March 11, 2021 were included. The entire Finnish population of 5.6 million was included. The positive influenza findings were stratified by age, and the monthly incidences per 100 000 persons in each age group were calculated. The population information was gathered from open‐access population statistics datasets maintained by the Finnish Population Center. Influenza seasons from August to July were compared. Due to the open‐access public data, no research permissions or ethical evaluation were obtained or needed.

## 3 RESULTS

**Figure 1:**
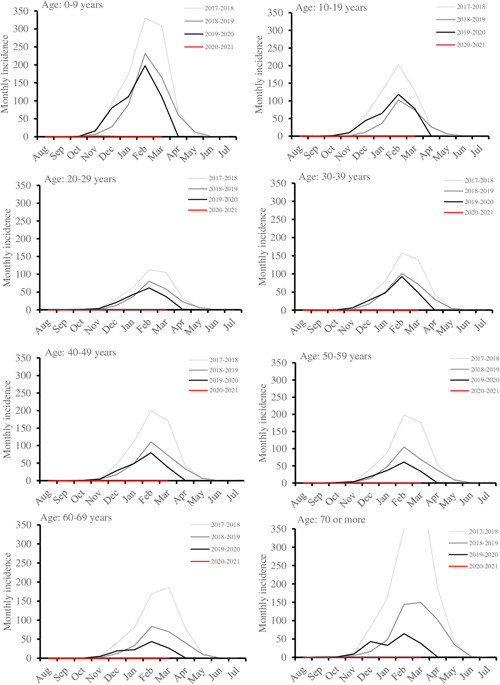
Monthly incidences per 100 000 persons of positive influenza A and B in Finland from August 2017 to March 2021. The data is stratified by age

Thus far, 41 influenza A and B cases have been reported in this season from August 2020 to March 2021, which adds up to an incidence of 0.9 per 100 000 person‐years. In the three preceding years, the numbers and corresponding incidences from August to March were 12 461 (282 per 100 000 person‐years) in 2019–2020, 15 276 (346 per 100 000 person‐years) in 2018–2019, and 33 659 (761 per 100 000 person‐years) in 2017–2018. The highest recorded monthly incidence during the 2020–2021 season was reported in November: 2.0 per 100 000 persons among 0–9 years old. Typically, the incidence peaks in February or March, and the highest recorded monthly peak during this study period was reported in March 2018 at 470 per 100 000 persons among residents aged over 70. The current influenza season has not begun among any age group (Figure 1).

## 4 DISCUSSION

The tight restrictions accompanying lockdown measures and school closures were effective in stopping the influenza season in March 2020, and the subsequent looser restrictions—including travel restrictions and the use of facial masks, without school closures—were effective in preventing the influenza season in winter 2020–2021.

The current numbers for this influenza season are a record low for Finland. A previous study in the United States reported similar findings of record low rates of influenza.^12^ The full season has not begun and will most likely not begin this winter, either in Finland or in the United States. Similar findings have not been recorded in the register since it was established in Finland in 1995. These findings are most likely due to the restrictions imposed to stop the spread of COVID‐19 because less than 50% of Finnish residents received influenza vaccination in 2020. Traveling restrictions have played an important role in the current low rates. Furthermore, the lockdown in March 2020 prevented the influenza seasons widely in the Southern hemisphere where they typically start in the spring. The low rates in the Southern hemisphere combined with the traveling restrictions have reduced the spreading potential of influenza and prevented the typical global circulation.

The restrictions strategy has changed many times during this pandemic, and the effectiveness in Finland had been good until novel SARS‐CoV‐2 variants caused a third wave of COVID‐19 in February 2021. This led to a second lockdown in March 2021. Before this, looser restrictions were in place from August 2020, with indoor social gatherings limited to 10–50 persons and restaurants closing at 10 p.m. Elementary schools and day‐care centers stayed open, and high schools and universities have been employing distance learning for most of the year. The policy has been that sick children should not attend day care or school, even when the symptoms are mild. Traveling has been avoided, although not restricted. Facial masks were promoted in August 2020 and have gained popularity, but are still not mandatory in public. All hospitals and healthcare workers have used masks since September in all interactions. The overall compliance with the restrictions has been good in Finland. In the United States, persons who associated SARS‐CoV‐2 with seasonal flu used less personal protection and did not change behavior.^13^ However, it is clear that the SARS‐CoV‐2 is more contagious and causes higher morbidity and mortality among adults than influenza.^14^ The severity of SARS‐CoV‐2 infection in children seems to be lower than in influenza.^15^

The strengths of this study are the nationwide Infectious Disease Register, which enables up‐to‐date surveillance of diseases on a large scale, and the high validity of this register. The main limitation of these results is the lack of testing numbers, as the denominator data would enable a test‐negative design. That data is, however, not recorded as the primary objective of the register is surveillance and not assessment (e.g., assessment of vaccination efficacy). It is possible that influenza cases may have been missed, as most tests currently being performed are for SARS‐CoV‐2. Especially children presenting with fever and mild respiratory symptoms might have been only tested for SARS‐CoV‐2 and not for other pathogens. However, for hospitalized respiratory infection patients, multiplex panel tests are performed, and because influenza results in hospitalization among the youngest and oldest age groups, the numbers presented in this study reflect an actual low incidence of influenza. Another limitation is the lack of vaccination status in the register, as vaccinations are reported to another register and this information is available earliest in September 2021.

Based on these results, it is clear that the current social restrictions were effective in preventing the spread of influenza. The harsh nonpharmaceutical interventions combined with the lockdown measures interrupted the influenza season in Finland in March 2020. Despite looser restrictions, alongside traveling restrictions and facial masks, failing to prevent the spread of the SARS‐CoV‐2 virus, these restrictions have proved to be effective against seasonal influenza. It remains of great interest to see how the 2021–2022 influenza season will unfold if the restrictions are abandoned, as well as the accuracy of the selected vaccination strains.

## CONFLICT OF INTERESTS

The authors declare that there are no conflict of interests.
